# ING5 inhibits cancer aggressiveness via preventing EMT and is a potential prognostic biomarker for lung cancer

**DOI:** 10.18632/oncotarget.3842

**Published:** 2015-04-15

**Authors:** Feng Zhang, Xutao Zhang, Jin Meng, Yong Zhao, Xinli Liu, Yanxia Liu, Yukun Wang, Yuhua Li, Yang Sun, Zhipeng Wang, Qibing Mei, Tao Zhang

**Affiliations:** ^1^ Key Laboratory of Gastrointestinal Pharmacology of Chinese Materia Medica of the State Administration of Traditional Chinese Medicine, Department of Pharmacology, School of Pharmacy, Fourth Military Medical University, Xi'an, China; ^2^ Department of Oncology, Tangdu Hospital, The Fourth Military Medical University, Xi'an, China; ^3^ Department of Pharmacy, No. 309 Hospital of PLA, Beijing, China; ^4^ Laboratory Animal Center, Fourth Military Medical University, Xi'an, China; ^5^ National Engineering Center for Biochip, Shanghai, China; ^6^ Department of Thoracic Surgery, Tangdu Hospital, Fourth Military Medical University, Xi'an, China

**Keywords:** ING5, lung cancer, proliferation, invasion, EMT

## Abstract

The proteins of the Inhibitor of Growth (ING) candidate tumor suppressor family are involved in multiple cellular functions such as cell cycle regulation, apoptosis, and chromatin remodeling. ING5 is the new member of the family whose actual role in tumor suppression is not known. Here we show that ING5 overexpression in lung cancer A549 cells inhibited cell proliferation and invasiveness, while ING5 knockdown in lung cancer H1299 cells promoted cell aggressiveness. ING5 overexpression also abrogated tumor growth and invasive abilities of lung cancer cells in mouse xenograft models. Further study showed that ING5 overexpression inhibited EMT indicated by increase of E-cadherin and decrease of N-cadherin, Snail and slug at mRNA and protein levels, which was accompanied with morphological changes. cDNA microarray and subsequent qRT-PCR validation revealed that ING5 significantly downregulated expression of EMT (epithelial to mesenchymal transition)-inducing genes including CEACAM6, BMP2 and CDH11. Clinical study by tissue microarray showed that nuclear ING5 negatively correlated with clinical stages and lymph node metastasis of lung cancer. Furthermore, high level of nuclear ING5 was associated with a better prognosis. Taken together, these findings uncover an important role for ING5 as a potent tumor suppressor in lung cancer growth and metastasis.

## INTRODUCTION

The Inhibitor of Growth (ING) proteins (ING1-ING5) have been identified and characterized as candidate tumor suppressors. All ING proteins share a highly conserved carboxy-terminal plant homeodomain (PHD) and are involved in the control of cell growth, senescence, apoptosis, DNA repair and chromatin remodeling [1-3]. ING5 is the new member of ING family and diverse function of ING5 has been reported since it was first identified by computational homology search. The pilot study of ING5 has shown that it physically interacts with p300 and p53, and overexpression of ING5 induces apoptosis in colorectal cancer cells [4]. Further, Doyon Y et al. [5] have revealed that ING5 associates with HBO1 and MOZ/MORF to form two distinct HAT complexes, which interact with the MCM helicase and are essential for DNA replication in human cells during S phase. Our previous study [6] also indicates that ING5 overexpression in MEF cells inhibits cell growth, induces a delay in S-phase progression and increases Fas-induced apoptosis, these functions are dependent on interaction with INCA1 which we have found to interact with and inhibit cyclin A1/CDK2 activity [7]. Recently, Mulder KW et al. [8] have identified ING5 as a component of genetic interacting network to control epidermal differentiation and protect epidermal stem cells from premature differentiation. These results indicate that ING5 plays an important role in the control of cell growth, apoptosis and differentiation, suggesting that abnormal function of ING5 may be linked to tumorigenesis and progression. However, the exact role of ING5 as a candidate tumor suppressor and the underlying mechanisms are largely unknown.

Aberrant expression of ING5 has been found in several tumors. Study on clinical samples finds mutation and downregulation of ING5 mRNA in oral squamous cell carcinoma [9]. In addition, we have found a significant reduction of ING5 expression in AML patients, further supporting a function of ING5 as a tumor suppressor [6]. Data from tissue array showed that ING5 translocation from the nucleus to the cytoplasm might be a critical event for carcinogenesis and tumor progression in human head and neck squamous cell carcinoma [10, 11]. Aberrant ING5 expression was also thought to contribute to pathogenesis, growth, and invasion of gastric carcinomas and colorectal cancer [12, 13].

However, the role of ING5 in lung cancer remains unknown. Lung cancer remains the leading cause of cancer mortality in the world with overall 5-year survival less than 15% due to the scarcity of effective tools for early detection and therapeutic strategies. Thus, finding specific biomarkers and revealing the molecular mechanisms of carcinogenesis and progression of lung cancer may lead to improvement of patient outcome.

In the current study, we have found that ING5 inhibits proliferation and invasion of lung cancer cells *in vitro* and *in vivo*. The underlying mechanisms may include its inhibition of EMT by downregulating EMT-inducing genes. By evaluating the correlation between ING5 expression and tumorigenesis, progression and prognosis of lung cancer with clinical samples, we have found that nuclear ING5 was inversely correlated with T status, clinical staging and lymphonode metastasis of lung cancer, and positively associated with a better overall survival. The current data provide insights into how ING5 functions as a tumor suppressor in lung cancer proliferation and metastasis, thus propose ING5 as a novel biomarker and potential therapeutic target for lung cancer.

## RESULTS

### Establishments of stable cell lines with ING5 overexpression and knockdown

**Figure 1:**
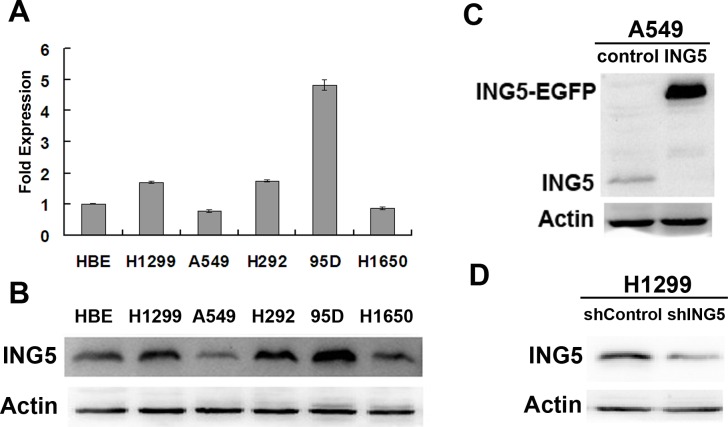
Establishment of ING5 overexpression and knockdown cell lines **A.** mRNA expression level of ING5 in lung cancer cell lines and normal human bronchial epithelial (HBE) cells by qRT-PCR. Data are shown as mean plus standard error of three independent experiments. **B.** Analysis of protein levels of ING5 in lung cancer cell lines and normal HBE cells by western blot. **C.** ING5-overexpressing A549 cells were generated using a lentiviral system. Empty vector-infected cells were used as control. ING5 protein levels in A549 control and A549 ING5 overexpression (ING5) cells were detected by western blot. Actin was used as an internal loading control. **D.** Stably ING5-depleted H1299 cells were generated using shING5-containing viruses. Cells infected with vector-scramble sequence were used as control (shControl). ING5 protein levels in H1299 shControl and H1299 ING5 knockdown (shING5) cells were determined by western blot.

ING5 mRNA and protein levels were detected in 5 lung cancer cell lines and one normal human bronchial epithelial (HBE) cells. ING5 is differentially expressed in the observed lung cancer cell lines. Compared with HBE cells, ING5 expression was higher in 95D and H1299 cells than HBE cells, while lower in A549 cells and H1650 cells (Figure 1A). Western blotting showed similar results (Figure 1B). To further investigate ING5 function in lung cancer, we chose A549 cells and H1299 cells to establish ING5 overexpression (ING5) stable cell line (Figure 1C) and ING5 knockdown (shING5) cell line (Figure 1D), respectively.

### ING5 inhibits proliferation, migration and invasion of lung cancer cells

**Figure 2:**
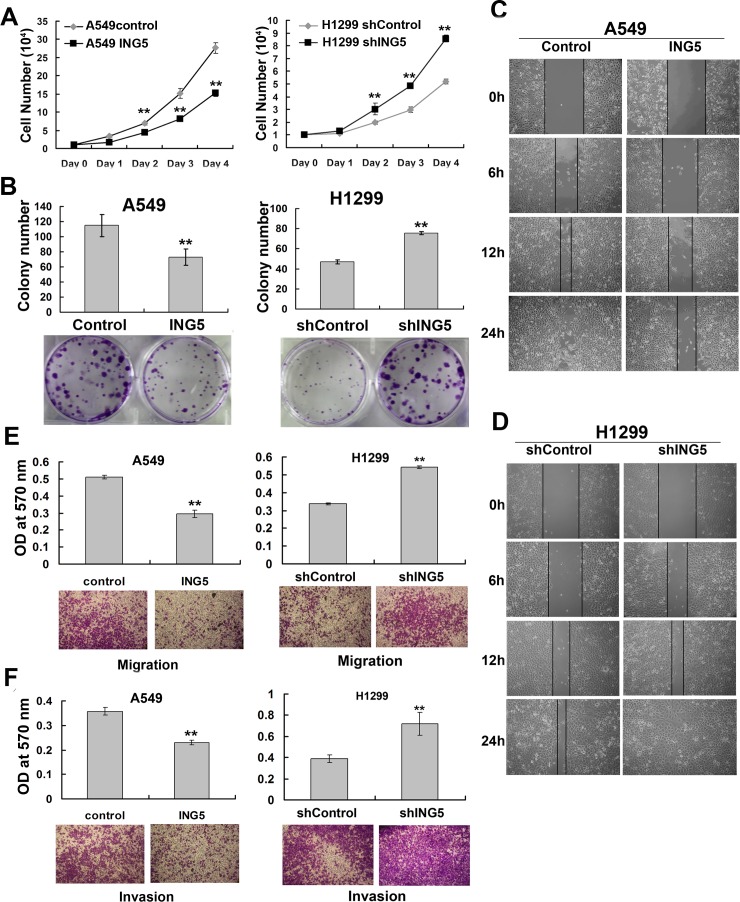
ING5 inhibits lung cancer proliferation, migration and invasion **A.** Effects of ING5 overexpression or knockdown on the proliferation of A549 and H1299 cells, respectively. Data are shown as mean plus standard error of three independent experiments. **P < 0.01 compared to corresponding control. **B.** Effects of ING5 overexpression or knockdown on colony formation of A549 and H1299 cells, respectively. Representative pictures are shown. Colony numbers were quantified. Data are shown as mean plus standard error of three independent experiments. **P < 0.01 compared to corresponding control. **C.** Wound healing assay was performed to show the effects of ING5 overexpression on migration of A549 cells. A scratch wound was made on cell surface and cells were photographed at 0h, 6h, 12h and 24h. Representative pictures are shown. **D.** Wound healing assay was performed to show the effects of ING5 knockdown on migration of H1299 cells. **E.** Effects of ING5 overexpression or knockdown on migration of A549 and H1299 cells, respectively. The migrated cells were photographed (100× magnification). Representative pictures are shown. The migrated cells were quantified by the absorbance of the crystal violet washed with 33% acetic acid. Data are shown as mean plus standard error of three independent experiments. **P < 0.01 compared to corresponding control. **F.** Effects of ING5 overexpression or knockdown on invasive abilities of A549 and H1299 cells, respectively. The invaded cells were photographed (100× magnification). Representative pictures are shown. The invaded cells were quantified by the absorbance of the crystal violet washed with 33% acetic acid from the cells that invaded the underside of the porous polycarbonate membrane. Data are shown as mean plus standard error of three independent experiments. **P < 0.01 compared to corresponding control.

Based on the ING5 overexpression and knockdown cell lines, the effects of ING5 on cell growth and invasive abilities were observed. The results showed that ING5 overexpression significantly inhibited cell proliferation and colony formation abilities of A549 cells, whereas these effects were promoted by ING5 knockdown in H1299 cells (Figure 2A, 2B). One characteristic of tumor metastasis is the increased ability of tumor cells migration. Our results showed that ING5 overexpression inhibited migration of A549 cells as assessed by would healing assay and transwell migration assay (Figure 2C, 2E). ING5 overexpression could also significantly prevent A549 cells from invading through Matrigel-coated polycarbonate filter in the transwell chamber (Figure 2F). As a confirmation, ING5 knockdown promoted cell migration and invasion in H1299 cells (Figure 2D, 2E, 2F). These results demonstrate that ING5 plays an anti-tumor role by inhibiting cell proliferation and invasion.

### ING5 inhibits tumor growth and invasion in mouse xenograft models

**Figure 3:**
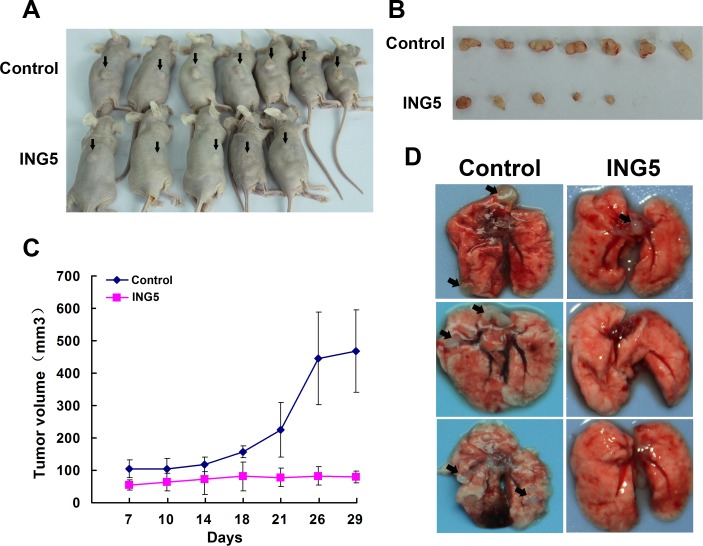
Overexpression of ING5 inhibits tumor growth and invasion of lung cancer cells *in vivo* **A.** Mice were injected subcutaneously with 5×10^6^ A549 control cells or A549 ING5 cells. At the end of experiment, mice were sacrificed and photographed (with arrows indicating tumors). **B.** Tumors isolated from the mice. **C.** Growth curves of tumor size. **D.** Effects of ING5 overexpression on lung tumor colonization in an intravenous mouse xenograft model. Representative gross images of lung show lung-metastasized tumors in both control and ING5 overexpression (ING5) groups of mice (with arrows indicating typical tumors) and tumor-free lungs in ING5 group mice at day 45 after tumor cell injection.

To define the role of ING5 in tumor growth ability *in vivo*, we established mouse xenograft models with A549 ING5 and A549 control cells by subcutaneous injection. ING5 overexpression significantly inhibited tumor formation and tumor growth. Tumor volumes were significantly decreased by ING5 overexpression (Figure 3A, 3B, 3C). We further assessed the effect of ING5 on tumor metastasis on the basis of an intravenous mouse xenograft models by injecting A549 ING5 and A549 control cells into tail veins of mice. Fourty-five days after injection, all mice (*n* = 5/5) that were injected with A549 control cells developed multiple tumors in bilateral lungs, whereas 2 out of 5 (*n* = 2/5) mice that were injected with A549 ING5 cells had lung tumors which were much less in quantity than control lung tumors (Figure 3D, Table 1).

**Table 1:** Lung tumor development in A549 control and ING5 groups in an intravenous xenograft mouse model

Groups	Mice (n)	Mice (n) with lung tumors	Average tumor numbers/lung
Control	5	5 (100%)	19
ING5	5	2 (40%)	3

### ING5 inhibits EMT by downregulating EMT-inducing genes

**Figure 4:**
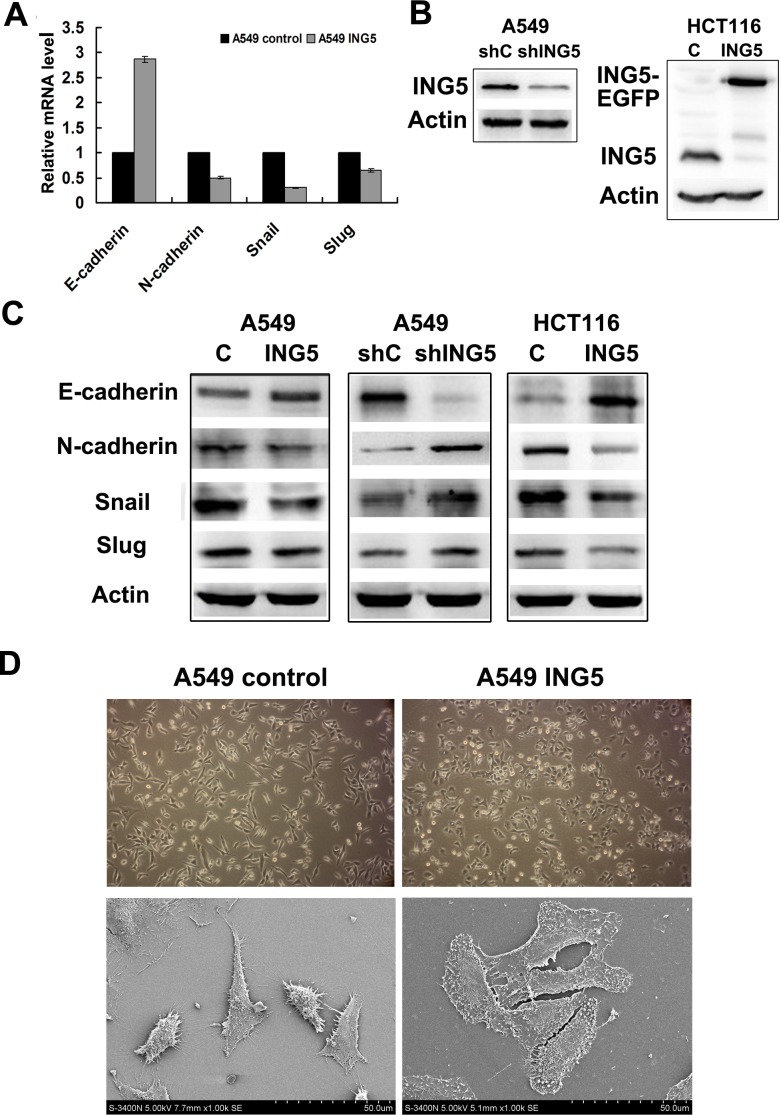
ING5 inhibits expression of EMT markers with morphological changes **A.** Effects of ING5 overexpression on expression of EMT markers by qRT-PCR. **B.** Stably ING5-depleted A549 cells were generated using shING5-containing viruses. Cells infected with vector-scramble sequence were used as control (shControl). ING5 protein levels in A549 shControl and A549 ING5 knockdown (shING5) cells were determined by western blot (Left). ING5-overexpressing HCT116 cells were generated using a lentiviral system. Empty vector-infected cells were used as control. ING5 protein levels in HCT116 control and HCT116 ING5 overexpression (ING5) cells were detected by western blot (Right). Actin was used as an internal loading control. **C.** Effects of ING5 overexpression or knockdown on protein expression of EMT markers by western blot. Actin was used as an internal loading control. **D.** ING5 overexpression induced cell morphological changes.: Micrographs of control and ING5-overexpressing A549 cells by light microscope (×100. upper) and by scanning electron microscope (×1000. lower).

The epithelial-to-mesenchymal transition (EMT) has been proved to play a critical role in driving tumor invasion and metastasis [14]. To investigate whether ING5 decreased cancer invasion by inhibiting EMT, we analyzed mRNA level of several EMT markers in ING5-overexpressing and control A549 cells. As shown in Figure 4A, qRT-PCR results revealed increased mRNA level of E-cadherin, an epithelial marker, and decreased mRNA levels of N-cadherin, a mesenchymal marker, SNAIL and Slug which are known EMT transcription factors [15], in ING5-overexpressing A549 cells. We further confirmed the changes of these EMT markers at protein level by western blot in ING5-overexpressing and -knockdown A549 cells, ING5-overexpressing colorectal cancer HCT116 cells, and their corresponding control cells (Figure 4B, 4C). However, no significant changes in Vimentin expression at both mRNA level and protein level were observed (not shown).

The changes of EMT markers were consistent with morphological alterations. As shown in Figure 4D, ING5-overexpressing A549 cells displayed more cobblestone-like cell morphology and cluster formation, the typical features of epithelial cells, compared with A549 control cells which exhibited a more mesenchymal phenotype with elongated shape and reduced cell–cell contact observed by inverted microscopy. Under scanning electron microscopy, the number of cell *filopodia*-like *protrusions* reduced significantly in ING5-overexpressing A549 cells. Taken together, these results indicate that overexpression of ING5 could inhibit EMT.

We then performed microarray analysis on ING5 overexpressing and control A549 cells. Differentially expressed gene profiles by ING5 overexpression in A549 cells were revealed by cDNA microarray. Genes upregulated or downregulated by 2 fold or more were listed, with 453 genes downregulated and 196 genes upregulated by ING5 overexpression (Supplemental files). qRT-PCR validation using ING5 control and overexpression cells (A549 ING5), and ING5 shControl and knockdown A549 cells (A549 shING5), were carried out for 30 selected genes which were known to be cancer-related genes out of genes downregulated by ING5 overexpression. The results confirmed 12 genes that were both significantly downregulated by ING5 overexpression and upregulated by ING5 knockdown (Table 2). Among the validated genes, CEACAM6, CDH11 and BMP2 are known EMT-inducing genes whose expressions changed over 5 fold by either ING5 overexpression or knockdown. These results suggest that ING5 may inhibit EMT by downregulating expression of EMT-inducing genes.

**Table 2:** List of ING5-downregulated genes by cDNA microarray analysis and qRT-PCR validation

Gene Symbol	cDNA array Ratio (control / ING5)	qRT-PCR Ratio (control/ING5)	qRT-PCR Ratio (shING5/shControl)
DEFB1	4.55	621.12	28.19
CDH11	5.00	568.18	57.8
TM4SF4	4.00	37.17	14.62
CEACAM6	3.45	30.67	31.4
GPC6	20.00	6.76	3.06
BMP2	4.35	5.18	8.78
MUC1	2.22	2.27	2.01
FAM40B	5.00	2.15	1.80
FN1	5.13	1.96	2.30
AGT	4.35	1.88	3.81
IL6	6.67	1.56	6.63
SNAI2	2.27	1.54	1.67

### High nuclear ING5 inversely correlates with lymphode metastasis and clinical stages and predicts better prognosis

**Figure 5:**
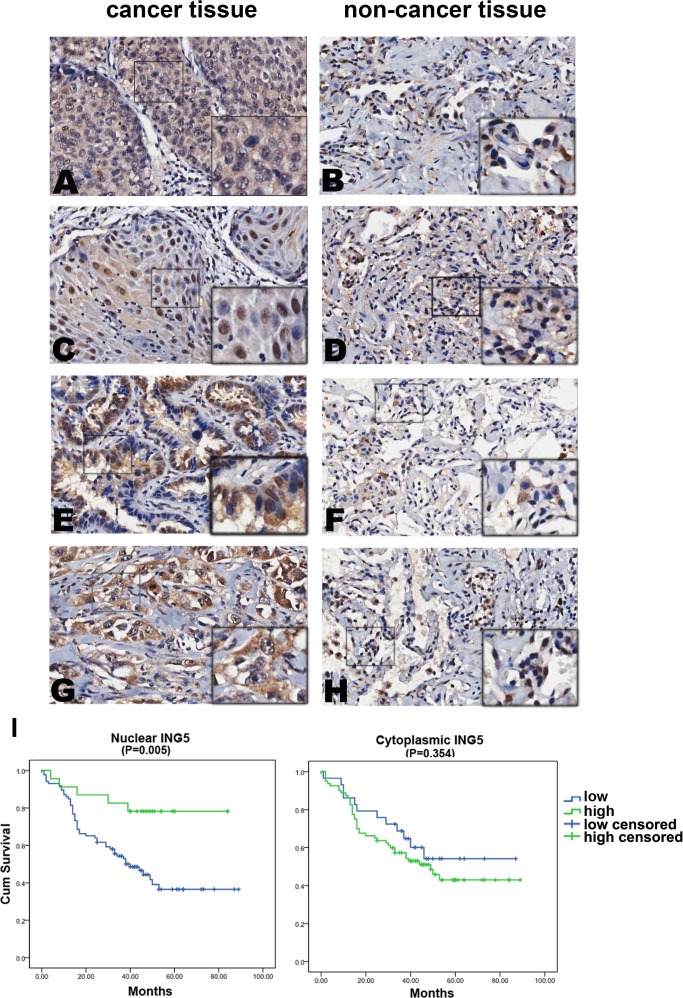
Nuclear ING5 in lung cancer tissues negatively correlates with clinical stages and lymphnode metastasis, and positively correlates with a better survival of lung cancer patients **A.**-**H.** Representative results of immunohistochemical staining of lung cancer specimens and corresponding adjacent non-cancerous lung tissues for ING5. ING5 was detected in both lung cancer tissues and normal tissues in cytoplasm (c) and nucleus (n). **A.** Squamous-cell carcinoma of lung cancer, ING5 staining score c/n (6/0), survival (14 days), N status (N1), staging (IIA); **C.** Squamous-cell carcinoma of lung cancer, ING5 staining score c/n (5/4.5), survival (68 days), N status (N0), staging (IA); **E.** Adenocarcinoma of lung cancer, ING5 staining score c/n (6/5), survival (59 days), N status (N0), staging (IA); **G.** Adenocarcinoma of lung cancer, ING5 staining score c/n (7/0), survival (3 days), N status (N2), staging (IIIB); **B**, **D**, **F** and **H.** adjacent non-cancerous lung tissues. Magnifications: ×200. **I.** Survival analysis of nuclear ING5 showed that patients with higher nuclear ING5 had better overall survival than patients with lower nuclear ING5 (P = 0.005); No correlation was found between patients' outcome and cytoplasmic ING5 level (P = 0.354).

In order to test whether the functional findings that ING5 inhibits lung cancer aggressiveness could be reflected in clinical samples, we evaluated the expression of ING5 by immunohistochemistry on tissue microarrays containing 150 lung tumors and corresponding non-cancerous lung tissue (Supplemental Table 1) and examined the correlation of ING5 expression with clinicopathological parameters in lung cancer patients. ING5 expression was detected in cytoplasm and nuclei in both lung cancer and non-cancerous tissues (Figure 5A-5H). The expression score of nuclear ING5 was significantly lower in tumor tissues than that in adjacent non-cancerous lung tissues (*P* = 0.042), while the cytoplasmic ING5 was significantly higher in lung cancer tissues than normal tissues (*P* = 0.000, Supplemental Table 2).

The correlation between ING5 expression and clinical parameters and prognosis of lung cancer was investigated. The results showed that the protein level of nuclear ING5 was negatively correlated with T staging (*P* = 0.02), lymph node metastasis (*P* = 0.006) and clinical staging (*P* = 0.024) of lung cancer patients (Table 3).

**Table 3:** Correlation of cytoplasmic and nuclear ING5 and clinicopathological features of lung cancer patients (n=150)

	n(%)	Cytoplasmic ING5			Nuclear ING5		
		low	High	p	low	high	p
Sex							
Female	38(25)	4(18)	34(82)	0.025	33(76)	5(24)	0.433
Male	112(75)	32(41)	80(59)		91(75)	21(25)	
Age							
≤60	55(37)	10(25)	45(75)	0.206	46(80)	9(20)	0.812
>60	95(63)	26(37)	69(63)		78(72)	17(28)	
Tumor size							
≤3cm	39(26)	9(23)	30(77)	0.876	30(77)	9(23)	0.272
>3cm	111(74)	27(24)	84(76)		94(85)	17(15)	
Differentiation/Grade							
Well+Moderately/G1+G2	89(59)	25(43)	64(57)	0.158	72(75)	17(25)	0.491
Poorly/G3	61(41)	15(25)	46(75)		52(75)	9(25)	
T status							
T1+T2	119(11)	29(29)	90(71)	0.836	94(65)	25(35)	**0.02**
T3+T4	31(68)	7(36)	24(64)		30(76)	1(24)	
N status (n=149)							
N0	78(52)	19(36)	59(64)	0.953	58(72)	20(28)	**0.006**
N1+N2+N3	71(24)	17(36)	54(64)		65(81)	6(19)	
M status							
M0	143(95)	34(35)	109(65)	0.773	107(75)	36(25)	0.216
M1	7(5)	2(43)	5(57)		7(86)	0(14)	
Stage							
I+II	98(36)	26(37)	72(63)	0.321	76(72)	22(28)	**0.024**
III+IV	52(29)	10(34)	42(66)		48(77)	4(23)	

Survival analysis was performed to identify whether ING5 has prognostic role in lung cancer patients. Significant association was observed between high level of nuclear ING5 and better overall survival (*P* = 0.005) (Figure 5I). We didn't observe a significant difference in the overall survival between patients with high level of cytoplasmic ING5 and those with low cytoplasmic ING5 (*P* = 0.354) (Figure 5I). These results suggest that ING5 may function as a nuclear tumor suppressor.

In order to see whether cancer and normal cell lines have the same expression pattern like tissues, we extracted cytoplasmic, nuclear and total proteins from lung cancer A549 and H1299 cells and normal human bronchial epithelial cell line HBE cells and detected ING5 protein level by western blot (supplemental Figure 1). The results showed that ING5 located in both cytoplasm and nuclei, with lower level in the cytoplasm than in the nuclei. There was little difference between HBE and lung cancer cell lines in cytoplasm ING5 level, unlike the expression pattern in tissues.

## DISCUSSION

ING5 belongs to the ING candidate tumor suppressor family. It has been reported that overexpression of ING5 in colorectal cancer cells resulted in reduced colony formation efficiency through interacting with P53 and P300 [4]. In line with the results, the present data also showed that ING5 overexpression could inhibit cell proliferation and colony formation in NSCLC A549 cells, while loss of ING5 promotes these processes in NSCLC cell line H1299 cells. Furthermore, we have found, for the first time, that ING5 could inhibit migration and invasion of lung cancer cells by both *in vitro* and *in vivo* study, suggesting a role for ING5 in preventing lung cancer metastasis. As A549 cells have wildtype p53 while H1299 cells have mutant p53, the current results from A549 and H1299 cells suggest that the anti-proliferative and anti-invasive effects of ING5 may not totally dependent on p53 function.

ING5 has been reported to be related to cell cycle progression and apoptosis. In MEF cells, we have observed a delay of S phase by ING5 overexpression [6]. However, little changes were found in cell cycle distribution between control and ING5 overexpression A549 cells (Supplemental Figure 3). It has been reported that ING5 functions as a cofactor of Tip60 for acetylation of p53 at K120, leading to activation of p53, expression of its target genes, BAX and GADD45, and subsequent apoptosis in response to DNA damage [16]. However, in current study under normal condition, ING5 overexpression was not able to induce significant apoptosis in A549 cells (Supplemental Figure 3). Further study is required for revealing the mechanisms underlying the anti-proliferation effect of ING5.

Epithelial–mesenchymal transition (EMT) plays a pivotal role in lung cancer metastasis [17,18]. Generally, increased motility and invasion are positively correlated with EMT, which is characterized by repression of epithelial markers and induction of mesenchymal markers. In ING5-overexpressed A549 cells, we detected an increased expression of E-cadherin which is a hallmark of EMT and often lost in metastatic tumors [19], while the expression of SNAIL, Slug and N-cadherin was downregulated, suggesting that ING5 inhibit lung cancer cell invasion by suppressing EMT. cDNA microarray screening and subsequent qRT-PCR verification identified several EMT-inducing genes which were downregulated by ING5, including CEACAM6 (Carcinoembryonic antigen-related cell adhesion molecule 6), CDH11 (cadherin-11) and BMP2 (bone morphogenetic protein 2), whose fold changes were over 5 by qRT-PCR validation with both ING5 overexpression and knockdown A549 cells. CEACAM6 has been considered a valid clinical biomarker and promising therapeutic targets in melanoma, lung, colorectal, and pancreatic cancers [20]. CEACAM6 overexpression has been identified as an independent predictor of poor overall survival in colorectal cancer patients [21]. Kim et al. have shown that attenuating CEACAM6 expression in LoVo cells impeded cell invasion by elevating E-cadherin promoter activity, suggesting its role in regulating metastasis [22]. CDH11 has been reported to be associated with increased metastatic ability of breast, prostate and colon cancers [23-25]. CDH11 contributes to pulmonary fibrosis by mediating transforming growth factor (TGF)-β-induced EMT and is induced by TGF-β, which is considered a prototypic inducer of EMT [26]. BMP2 is a member of TGF-β superfamily and is also involved in EMT formation [27]. BMP2-induced lung cancer migration is associated with up-regulation of Runx2 which recruits p300 and in turn enhances histone acetylation, increases Snail expression, and decreases E-cadherin [28]. The stimulation of BMP2 in gastric cancer cells induces a full EMT characterized by Snail induction, E-cadherin delocalization and down-regulation, and up-regulation of mesenchymal and invasiveness markers [29]. PI3K/Akt-NF-kappaB signaling may promote prostate cancer (PC) bone metastasis in part by regulating transcription and activation of BMP2 and subsequent phosphorylation of Smad1/5/8 which are critical downstream targets of BMP2 signaling [30]. The current results suggest that ING5 could inhibit lung cancer metastasis by preventing EMT through downregulating EMT-inducing genes.

It is interesting how ING5 downregulates such a group of EMT-related genes. ING5 has been known to contribute to form two distinct HAT complexes by association with HBO1 and MOZ/MORF through the conserved plant homeodomain (PHD) finger motif, and thus has been defined as a transcription coactivator and regulates gene transcription through chromatin acetylation [5, 33-33]. However, the concise mechanisms for ING5 as a gene transcription regulator needs to be further investigated.

The present study by tissue array showed that ING5 was found in normal lung and lung cancer tissues in both cytoplasm and nuclei, and lower nuclear ING5 level was seen more frequently in lung cancer tissues than in normal lung tissues. Furthermore, nuclear ING5 was negatively correlated with lymph node metastasis and cancer staging, and was closely linked to better prognosis of lung cancer patients. These results are in line with that from gastric cancer patients [12] and partly support our data from lung cancer cell lines that ING5 functions a tumor suppressor by preventing lung cancer metastasis.

Unexpectedly, much higher cytoplasmic level of ING5 was found in lung cancer tissues than that in normal lung tissues, suggesting that increased ING5 expression in cytoplasm could serve as a biomarker for lung cancer and may contribute to lung carcinogenesis, and that ING5 translocation from nuclei to cytoplasm might be one reason for increased ING5 in cytoplasm where it lost anti-tumor function. The function of cytoplasmic ING5 and the mechanisms by which it correlates with lung cancer need to be further defined. These results suggest that the effects of ING5 depend on its intracellular location and cytoplasmic translocation of ING5 contributes to carcinogenesis and progression of lung cancer, like tumor suppressor p27^Kip1^, which serves as a cytoplasmic oncogene in melanoma by relocalization [34]. These results also indicate that the anti-tumor effects of ING5 are greatly associated with its role of transcription regulation, which depends on its nuclear localization where it binds to specific modified histones.

Recent study suggests that ING family proteins may play dual roles, as tumor suppressors or oncogenes, under different cellular conditions [35]. Based on the current results we also propose that ING5 may play dual roles depending on its cellular location. The mechanisms mediating the translocation of ING5 during lung cancer initiation and progression are unknown, which may include protein-protein interaction and post-translational modifications (PTMs). Our previous study [6] on MEF cells have shown that ING5 inhibits cell growth by interacting with INCA1 which could associate with cyclin A1/CDK2 and inhibits CDK2 activity, however, it promotes cell growth when INCA1 is absent. These results suggest that the interaction with other proteins may greatly influence ING5 function. Whether the oncogenic function of ING5 without INCA1 association may lead to cytoplasmic location of ING5 deserves further investigation. Uncovering the mechanisms would make it possible to reverse the cytoplasmic translocation of ING5 and retain its normal function.

Besides, the current study also raises another question for future research. Our results show that ING5 expression at both mRNA and protein level differs in a broad variety in lung cancer cell lines. To our surprise, the highest expression level of ING5 was detected in lung cancer cell line 95D with high metastatic potential, which seems to contradict our conclusion that ING5 prevents proliferation and invasion of lung cancer cells. The underlying mechanisms need to be further defined. However, it would be important and interesting for the first step, to investigate more cell lines to demonstrate a broader expression panel of ING5 in different cell lines with comparison of their proliferative and invasive abilities.

In conclusion, our results demonstrate that ING5 could inhibit EMT and aggressiveness of lung cancer cells by downregulating EMT-inducing genes. As early and frequent metastasis is the major cause for high mortality of lung cancer patients, the current data propose ING5 as a biomarker and potent target for anti-metastasis therapy of lung cancer. The regulatory mechanisms mediating ING5 translocation and gene expression regulation need to be further investigated.

## MATERIALS AND METHODS

### Cell culture

Human lung cancer cell lines (A549, H1299, H1650, H292 and 95D) and human colorectal cancer cell line (HCT116) were purchased from the Type Culture Collection of the Chinese Academy of Sciences, Shanghai, China. Normal human bronchial epithelial cell line *HBE cells* were purchased from Genechem, Shanghai, China. These cells were grown in Dulbecco's modified Eagle's medium (DMEM, Gibco, USA) supplemented with 10% fetal bovine serum (HyClone, USA), 10mg/ml antibiotics (penicillin and streptomycin) and 2 mmol/L L-glutamine at 37°C under 5% CO_2_ and saturated moisture. human bronchial epithelial cell line *HBE* human bronchial epithelial cell line *HBE*

### Quantitative RT-PCR

Total RNA was isolated using TRIzol reagent (Invitrogen, CA, USA). RNA (1μg) was reverse-transcribed using random primers and MMLV reverse transcriptase (Promega, Madison, WI) following the manufacturer's protocol. Quantitative RT-PCR was performed starting with 1 μl cDNA, sequence-specific primers, and SYBR Green Realtime PCR Master Mix (Applied Biosystems) at the following thermal conditions: 95°C for 10 min and 40 cycles at 95°C for 15 s and 60°C for1 min. Expression of the housekeeping gene glyceraldehyde-3-phosphate-dehydrogenase (GAPDH) was used to normalize the amount of cDNA between different samples. Primers used were listed in supplemental file. All samples were performed in triplicate for three times.

### Western blot

Cells were lysed in lysis buffer containing 150mM NaCl, 1% NP40, 0.5% deoxycholic acid, 0.1% SDS, 50mM Tris (pH 8.0), and 1:25 protease inhibitor cocktail for total protein. Nuclear and cytosolic protein were extracted using Nuclear/cytosol fractionation kit (BioVision Incorporated, CA). Protein concentrations of the lysates were determined by the Bradford protein assay system (Bio-Rad, Hercules, CA). Equal amounts of protein (30μg protein each lane) were separated by SDS-PAGE, transferred to nitrocellulose membranes (Hybond C, Amersham, UK). Immunoblots were blocked with 5% skim milk in TBS/Tween 20 (0.05%, v/v) for 1 hour at RT. The membrane was incubated with primary antibody overnight at 4°C. Primary antibodies used include ING5 antibody (Proteintech Group, Inc. IL), H2AX antibody (Santa Cruz Biotechnology Inc.), antibodies for E-cadherin, Snail and N-cadherin (Abcam) and β-actin (Actin) antibody (Sigma). The membrane was incubated with corresponding secondary antibody conjugated with horseradish peroxidase (Sigma) (1:5000) at RT for 1h. The blots were developed using an enhanced chemiluminescence western blotting detection system (Amersham Bioscience, UK).

### Constructs and establishment of stable cell lines

To generate stable ING5 overexpression cells, A549 cells were infected with GV218-EGFP-ING5 lentivirus construct (Genechem). Single-cell clones were isolated by 5μg/ml Puromycin for 48h followed by 1μg/ml puromycin treatment. Empty vector-infected cells were used as control. Overexpression of ING5 was confirmed by western blot with a molecular weight of 52kD. To generate stable ING5 knockdown cells, A549 cells or H1299 cells were infected with pLVX-shING5 virus construct (Biowit Technologies LTD). Cells were selected in 5μg/ml Puromycin containing medium. Cells infected with vector-scramble sequence were used as control (shControl). Down-regulated protein level of ING5 was confirmed by western blot.

### Proliferation assay

Cells were seeded in triplicate in 24-well plates at a density of 1×10^4^ cells/well. Cell numbers were counted every 24h after cell adhesion over a 4-day period.

### Colony formation assay

300 cells were seeded in 6-well plates and incubated for 15 days when colonies were visible. Crystal violet staining was performed and the number of colonies was counted.

### Wound-healing assay

Cells were seeded in 6-well plates at a density of 4×10^5^ cells/well. Once the cells reached 90% confluence, a wound area was carefully created by scraping the cell monolayer with a sterile 200 μl pipette tip, from one end to the other end of the well. The detached cells were removed by washing with PBS. Cells migrated to the wounded region were observed by Olympus CK-2 inverted microscope and photographed (100× magnification) at 0h, 6h, 12h and 24h. The experiments were performed in triplicate.

### Transwell migration and invasion assay

For the migration assay, 5×10^4^ cells were suspended in serum-free medium and plated on chambers (Corning Costar, NY, USA) that were not coated with Matrigel. For the invasion assay, the upper chamber was precoated with Matrigel (BD Bioscience, CA, USA) according to the manufacturer's protocols before 5×10^4^ cells in serum-free RPMI-1640 were added to the chamber. For both assays, medium containing 10% FBS was added to the lower chamber as a chemoattractant. The cells were incubated at 37 °C in the incubator supplemented with 5% CO_2_ for 12h (migration) or 16h (invasion). Non-invasive cells in the upper chamber were removed by wiping with a cotton swab, and invasive cells were fixed with 4% formaldehyde in PBS and were stained with 1% crystal violet in 2% ethanol. Cells in the lower surface of the filter were photographed under a light microscope (100× magnification). The inserts were washed with 33% acetic acid. Absorbance of washing buffer at 570 nm was determined for each well using a microplate reader. Cell-free inserts containing only medium had been included in duplicate throughout each experiment as OD background controls. Reported OD data represent average background-corrected values ± SD obtained from three independent experiments in duplicate.

### Xenograft studies

Male athymic nude mice (4 weeks old) were bought from Experimental Animal Center of Fourth Military Medical University. All animal procedures were performed in according with protocols approved by the Animal Care and Use Committee of Fourth Military Medical University. Mice were injected subcutaneously with 5×10^6^ A549 control cells or A549 ING5 cells. Tumor size was monitored with calipers twice a week. Tumor volume was estimated as (D^2^×d) / 2, where D is the large diameter and d is the small diameter of the tumor. For the intravenous mouse model, mice were injected with 5×10^6^ A549 control cells or A549 ING5 cells through teil vein and mice were sacrificed at day 45 after injection and lungs were inspected for tumor formation.

### Scanning electron microscopy

Cells were seeded on glass coverslips. After attachment, cells were fxed with 2.5% glutaldehyde for 45 min and 1% osmium tetroxide for 30 min followed by step-wise ethanol dehydration. After the step of critical point drying, slides were coated with gold-palladium. Images were scanned with scanning electron microscope (S-3400N, HITACHI) and captured under ×1,000 magnifcations.

### Gene expression analysis by microarray

Total RNA was extracted and purified using mirVana^TM^ miRNA Isolation Kit (Ambion, Austin, TX, US) following the manufacturer's instructions and checked for a RIN number to inspect RNA integration by an Agilent Bioanalyzer 2100 (Agilent technologies, Santa Clara, CA, US). Total RNA were amplified, labeled and purified by using GeneChip 3′IVT Express Kit (Affymetrix, Santa Clara, CA, US) following the manufacturer's instructions to obtain biotin labeled cRNA. Samples were analyzed with GeneChip^®^ PrimeView™ Human Gene Expression Array (Cat#901837, Affymetrix Inc.). Array hybridization and wash was performed using GeneChip○RR Hybridization, Wash and Stain Kit (Affymetrix, Santa Clara, CA, US) in Hybridization Oven 645 and Fluidics Station 450 following the manufacturer's instructions. Slides were scanned by GeneChip^®^ Scanner 3000 (Affymetrix, Santa Clara, CA, US) and Command Console Software 3.1 (Affymetrix, Santa Clara, CA, US) with default settings. Raw data were normalized by RMA algorithm, Gene Spring Software 11.0 (Agilent technologies, Santa Clara, CA, US). The ratio between mRNA levels in A549 ING5 and in A549 control cells was calculated and only the upregulation and downregulation by 2 fold or more in gene expression was considered significantly.

### Tissue microarray

The tissue microarrays (TMAs) used in this study were obtained from National Engineering Center for Biochip at Shanghai. All tissues investigated in the present study were obtained from 150 lung cancer patients undergoing complete surgical resection of the lung tumor as an initial treatment between July 2004 and April 2009. The specimens were fixed in 10% formalin. Paraffin-embedded tumor specimens and paired adjacent non-tumor specimens were collected for histological examination. All patients were followed up until August 2012. Two histological tumor subtypes including adenocarcinoma (75 patients) and squamous cell carcinoma (75 patients) were examined. Detailed clinical and pathologic information of patients were summarized in Supplemental Table 1.

This study was performed under the approval of the Clinical Research Ethics Committee of the Fourth Military Medical University. Written informed consent was signed by the patients to allow for the research to be undertaken.

### Immunohistochemistry of TMA

TMA slides were routinely deparaffinized and rehydrated. Antigen retrieval was performed by heating the samples at 95°C for 30 min in 10 mM sodium citrate (pH 6.0). Endogenous peroxidase activity was blocked by immersing the samples in 3% hydrogen peroxide for 5 min. Non-specific antibody binding sites were then blocked by incubating in 5% bovine serum albumin (BSA) for 30 min. Samples were incubated with anti-ING5 antibody (1:25 dilution) at 4°C over night and then incubated with a biotin-labeled secondary antibody followed by streptavidin-peroxidase. The color reaction was developed in 3, 3′-diaminobenzidine for 5 min and counterstained with hematoxylin.

### Evaluation of immunohistochemical staining

The intensity of ING5 immunostaining was scaled as no stain (0), weak staining (1+), moderate staining (2+), or strong staining (3+). The percentage of positive cells was scored as follows: 0, 0%; 1, 1-20%; 2, 21-40%; 3, 41-60%; 4, 61-80%; 5, 81-100%. A final score was obtained by multiplying both intensity and percentage ranging from 0 to 8. Since most stained tissues showed positive expression of ING5, the immunohistochemical results of ING5 were grouped into two categories: low expression (0-3) and high expression (3.1-8).

### Statistical analysis

Two groups of data were analyzed by t test. The differential level of cytoplasmic or nuclear ING5 between normal lung tissue and lung cancer tissue was evaluated with Paired-Samples t test. Association of cytoplasmic and nuclear ING5 in both cancer and non-cancerous tissues with clinicopathological features was evaluated using Mann-whitneyU and Kruskal-Wallish methods. Relationships between ING5 expression and clinicopathological parameters were statistically analyzed using Spearman's rank correlation coefficient. Survival curves were estimated using the Kaplan-Meier method and compared by the log-rank test. Overall survival was determined from the date of surgery and histological diagnosis to the time of last contact or death due to any cause. All of the statistical tests were 2-sided. P<0.05 was regarded as statistically significant. All data were analyzed with SPSS 17.0 software program.

## SUPPLEMENTAl MATERIAL FIGURES AND TABLES


