# Supplementary material for: ING5 inhibits cancer aggressiveness via preventing EMT and is a potential prognostic biomarker for lung cancer
Source: Oncotarget. 2015 Apr 15;6(18):16239–52. doi: 10.18632/oncotarget.3842 (PMC4599267; doi:10.18632/oncotarget.3842)
Supplement: Supplementary file 1 [file oncotarget-06-16239-s001.pdf]

ING5 inhibits cancer aggressiveness via preventing EMT and is a potential prognostic biomarker for lung cancer

Supplementary Material

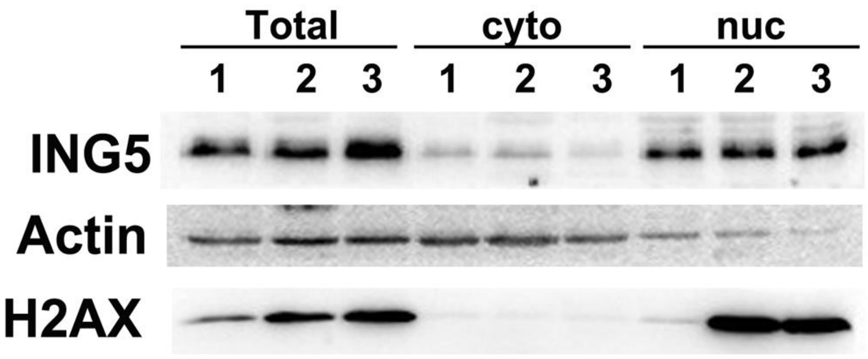

Supplemental Figure 1: ING5 expression was detected in both cytoplasm and nuclei in lung cancer A549 and H1299 cells and normal human bronchial epithelial cell line HBE cells. Cyto: cytoplasm; nuc: nuclei. Actin was used as an internal loading control for total and cytoplasmic protein, while H2AX was used as an internal loading control for nuclear protein. 1: HBE cells; 2: A549 cells; 3: H1299 cells.

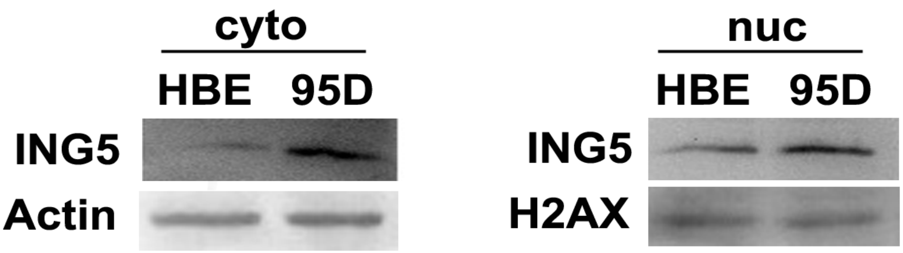

Supplemental Figure 2: ING5 protein level was detected in both cytoplasm and nuclei in lung cancer 95D cells and normal human bronchial epithelial cell line HBE cells. Cyto: cytoplasm; nuc: nuclei. Actin was used as an internal loading control for cytoplasmic protein, while H2AX was used as an internal loading control for nuclear protein.

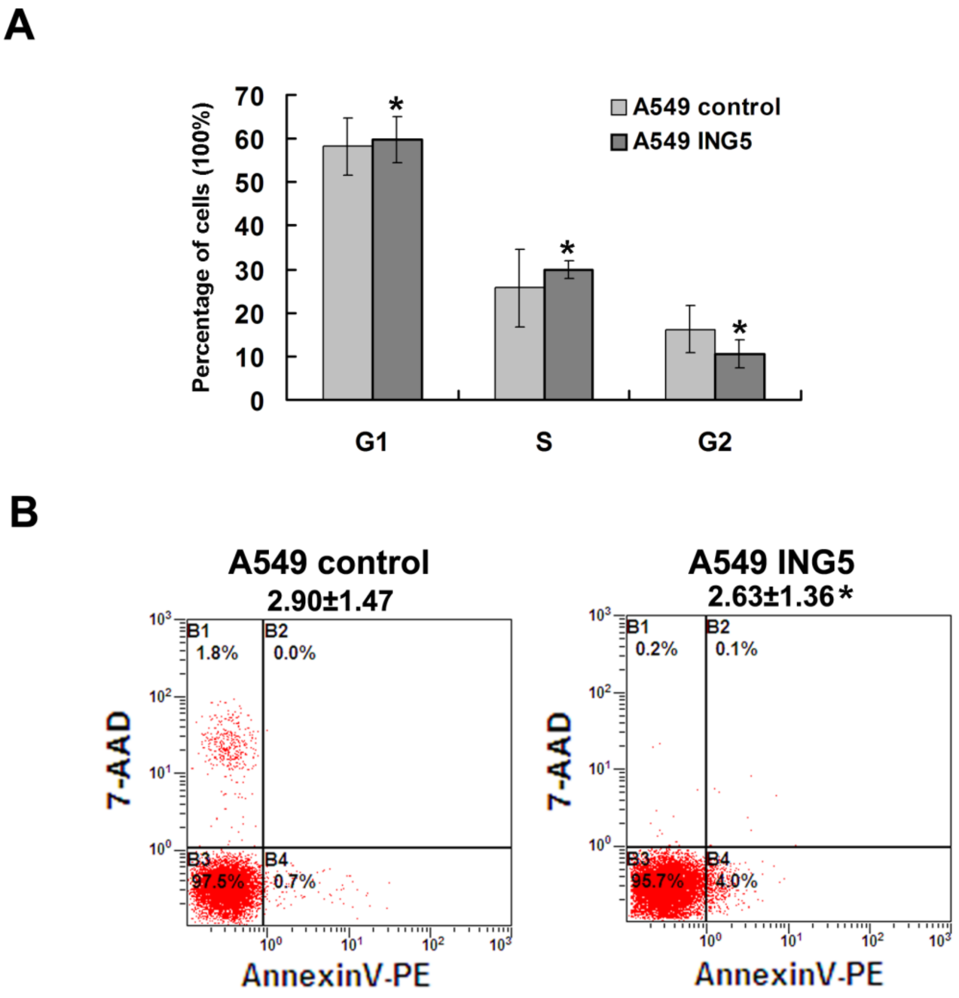

Supplemental Figure 3: Cell cycle and apoptosis analyzed by flow cytometry with control and ING5 overexpressing A549 cells. (A) Cell cycle distribution showed no significant changes by ING5 overexpression. (B) Apoptosis was not influenced by ING5 overexpression. Data are shown as mean plus standard error of three independent experiments. *P>0.05 compared to A549 control.

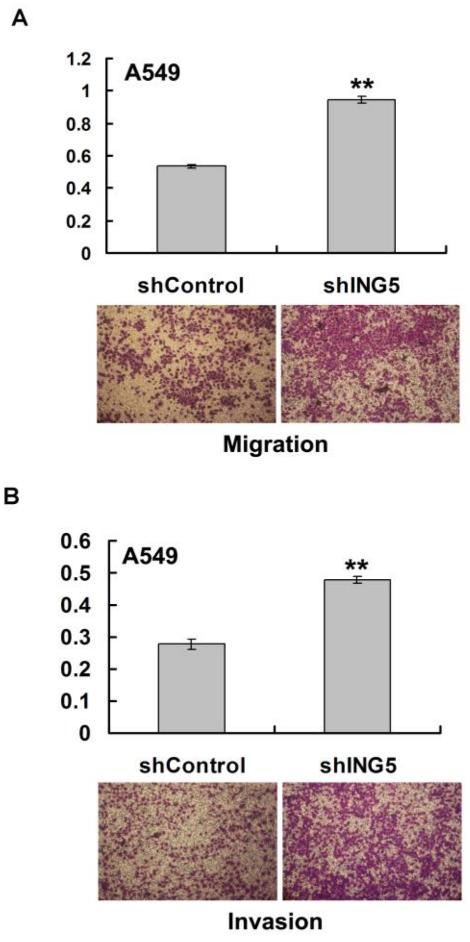

Supplemental Figure 4: ING5 knockdown promotes migration and invasion of A549 cells. **(**A) Effects of ING5 knockdown on migration of A549 cells. The migrated cells were photographed (100× magnification). Representative pictures are shown. The migrated cells were quantified by the absorbance of the crystal violet washed with 33% acetic acid. Data are shown as mean plus standard error of three independent experiments. **P<0.01 compared to shControl. (B) Effects of ING5 knockdown on invasive abilities of A549 cells. The invaded cells were photographed (100× magnification). Representative pictures are shown. The invaded cells were quantified by the absorbance of the crystal violet washed with 33% acetic acid from the cells that invaded the underside of the porous polycarbonate membrane. Data are shown as mean plus standard error of three independent experiments. **P<0.01 compared to shControl.

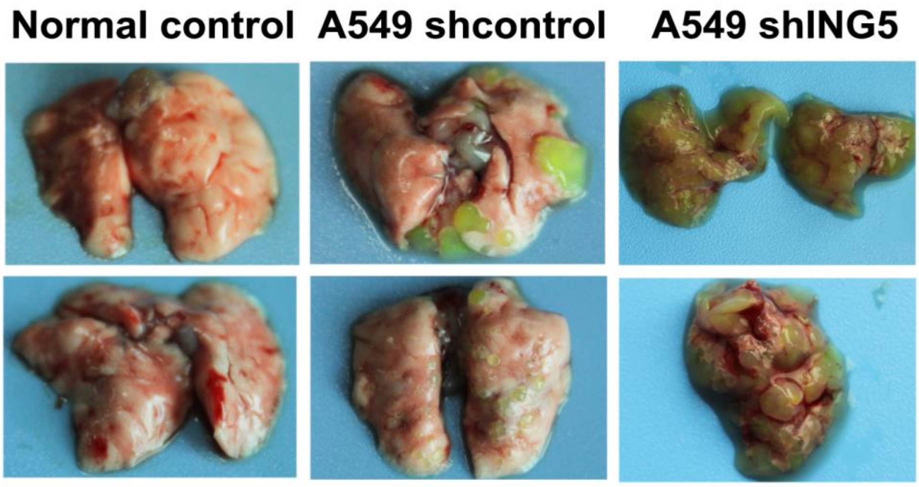

Supplemental Figure 5: Effects of ING5 knockdown on lung tumor colonization in an intravenous mouse xenograft model. ING5 knockdown promotes tumor invasion of lung cancer cells in vivo. Representative gross images of lung show lung-metastasized tumors in both shControl and shING5 groups of mice at day 50 after tumor cell injection. Normal control indicates normal lungs from mice without tumor cell injection.

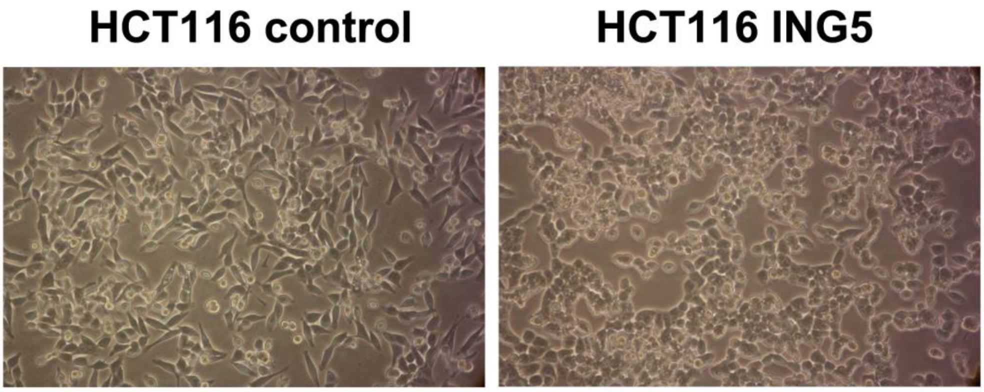

Supplemental Figure 6: ING5 overexpression induced cell morphological changes in HCT116 cells. Micrographs of control and ING5-overexpressing human colorectal cancer HCT116 cells by light microscope（×100).

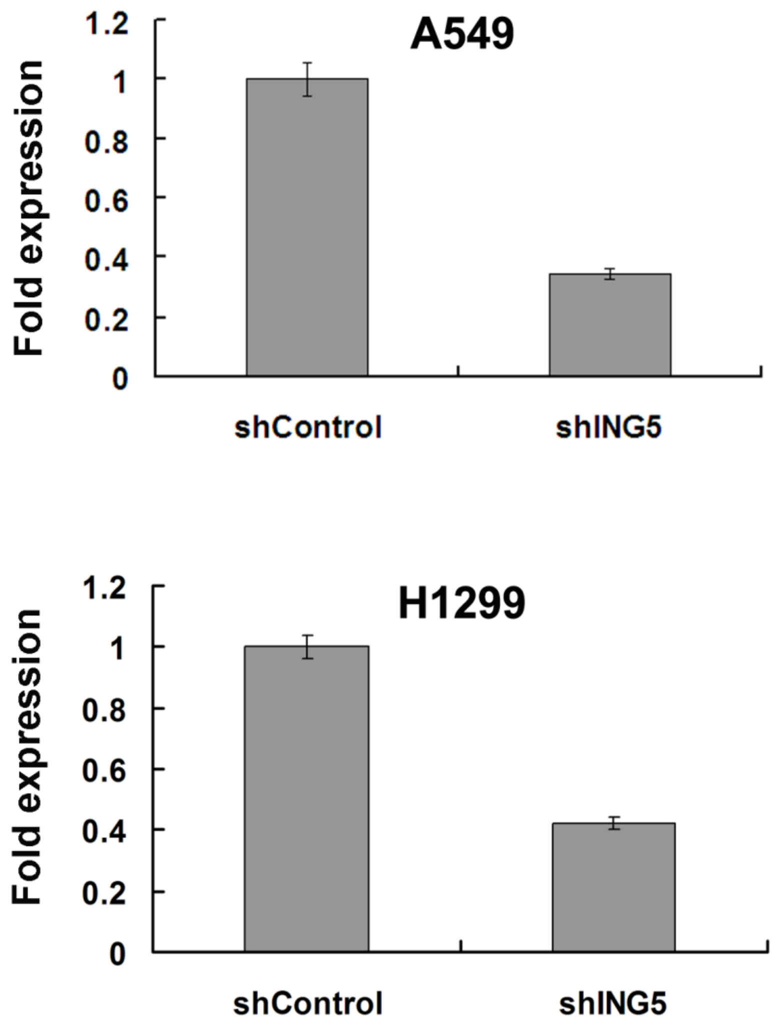

Supplemental Figure 7: ING5 knockdown efficiency measured by qRT-PCR. mRNA levels of ING5 from shControl and shING5 A549 and H1299 cells were measured by qRT-PCR. Data are shown as mean plus standard error of three independent experiments.

Supplemental Table 1: Clinical and pathologic characteristics of patients

Characteristics	n	(%)
All	150	100
**Sex**		
Female	38	25%
Male	112	75%
**Age** (n=149)		
≤60	55	37%
＞60	94	63%
**Tumor siz**e		
≤3cm	39	26%
＞3cm	111	74%
**Histological type**		
Adenocarcinoma	75	50%
Squamous cell carcinoma	75	50%
**Differentiation/Grade**		
Well+Moderately/G1+G2	89	60%
Poorly/G3	61	40%
**T status**		
T1+T2	119	79%
T3+T4	31	21%
**N status** (n=149)		
N0	78	52%
N1+N2+N3	71	48%
**M status**		
M0	143	95%
M1	7	5%
**Stage**		
Ⅰ+Ⅱ	98	65%
Ⅲ+Ⅳ	52	35%

Supplemental Table 2: Differential analysis of cytoplasmic or nuclear ING5 expression in cancer and adjacent normal tissues.

		Expression score (Mean±Std. Deviation)	P value*
Cytoplasmic ING5	Normal tissue	1.0422±1.0982	0.000
	Cancer	5.0000±1.9793	
Nuclear ING5	Normal tissue	1.7821±1.0624	0.042
	Cancer	1.4983±1.6950	

* The paired t-test was used for statistic analysis and P<0.05 is considered significant.

Supplemental Table 3: Relationship between the expression of ING5 and clinicopathological parameters.

Parameters		Cytoplasmic ING5	Nuclear ING5
**Sex**	r	0.184	-0.064
n=150	P	**0.024**	0.435
**Age**	r	-0.104	0.019
n=150	P	0.207	0.813
**Tumor size**	r	-0.013	-0.090
n=150	P	0.876	0.274
**Differentiation/Grade**	r	0.116	-0.056
n=150	P	0.159	0.493
**T status**	r	0.017	-0.190
n=150	P	0.837	**0.020**
**N status**	r	0.005	-0.226
n=149	P	0.953	**0.006**
**M status**	r	-0.024	-0.101
n=150	P	0.774	0.217
**Stage**	r	0.081	-0.186
n=150	P	0.322	**0.023**

r: correlation coefficient
